# Racial and ethnic differences in cervical cancer screening barriers and intentions: The My Body My Test-3 HPV self-collection trial among under-screened, low-income women

**DOI:** 10.1371/journal.pone.0274974

**Published:** 2022-10-13

**Authors:** Erica E. Zeno, Noel T. Brewer, Lisa P. Spees, Andrea C. Des Marais, Busola O. Sanusi, Michael G. Hudgens, Sarah Jackson, Lynn Barclay, Stephanie B. Wheeler, Jennifer S. Smith

**Affiliations:** 1 Department of Epidemiology, Gillings School of Global Public Health, University of North Carolina at Chapel Hill, Chapel Hill, NC, United States of America; 2 Department of Health Behavior, Gillings School of Global Public Health, University of North Carolina at Chapel Hill, Chapel Hill, NC, United States of America; 3 Lineberger Comprehensive Cancer Center, University of North Carolina, Chapel Hill, NC, United States of America; 4 Department of Health Policy and Management, Gillings School of Global Public Health, University of North Carolina at Chapel Hill, Chapel Hill, NC, United States of America; 5 Department of Biostatistics, Gillings School of Global Public Health, University of North Carolina at Chapel Hill, Chapel Hill, NC, United States of America; 6 American Sexual Health Association, Research Triangle Park, NC, United States of America; Kaiser Permanente Washington, UNITED STATES

## Abstract

Under-screened women are more likely to be diagnosed with invasive cervical cancer at later stages and have worse survival outcomes. Under- or un-insured women, low-income women, and minoritized groups face barriers to screening. Intention to screen is an indicator of future screening behavior, yet is understudied among low-income, under-screened women. Participants were 710 low-income, uninsured or publicly insured women ages 25–64 years in North Carolina who were not up to date on cervical cancer screening according to national guidelines. Participants were asked about barriers to screening and intention to screen. We estimated reported barriers to cervical cancer screening stratified by race and ethnicity (categorized as White, Black, and Hispanic) and assessed predictors of intention to screen. Sixty-one percent of all participants reported 5 or more barriers to screening. The most commonly reported reasons for not getting screened were lack of insurance (White: 71%, Black: 62%, Hispanic/Latina: 63%) and cost (White: 55%, Black: 44%, Hispanic/Latina: 61%). Women were more likely to have an intention to screen if they reported “it was not hard to get screening” (OR: 1.47 (1.00, 2.15)). Older women reported being less likely to intend to screen. Black women reported being more likely to intend to screen than White women. Lack of health insurance and cost were frequently reported barriers to cervical cancer screening. Increasing knowledge of affordable clinics and expanding access to Medicaid may reduce barriers and increase cervical cancer screening uptake.

## Introduction

With early detection through regular screening, cervical cancer is both detectable and preventable [1]. Increased uptake of screening tests has been largely responsible for the decrease in cervical cancer incidence and mortality in the United States [2]. The U.S. Preventive Services Task Force (USPTF) currently recommends that women ages 21 to 65 should receive a Pap test every 3 years, or, for women 30 or older, testing for oncogenic human papillomavirus (HPV) infection as a primary screening test or in conjunction with Pap testing every 5 years [3]. Women who do not receive regular screenings are at a higher risk of cervical cancer, and tend to be diagnosed at later stages with worse survival outcomes [4].

Cervical cancer screening uptake varies by socioeconomic factors including income, and education level [5–9]. The National Health Interview Survey estimates that in 2019, only 64% of women whose income was <200% of the federal poverty level were up-to-date with cervical cancer screening compared to 78% of women whose income was above 200% of the federal poverty level [10]. Fifty-nine percent of women with less than a high school education were up-to-date on screenings compared to 67% with a high school degree and 78% of women with education beyond high school [10]. These socio-economic disparities in screening lead to excess cervical cancer morbidity and mortality in under-screened, underserved groups [4].

Barriers to screening are a major deterrent to increasing positive health behaviors according to the Health Belief Model [11]. Barriers to cervical cancer screening include fear of cancer or of the screening procedure [12–16], embarrassment [13, 15, 16], lack of knowledge [12, 13, 17], having a male physician as the service provider [12, 15, 16], cost [15, 16, 18], having to take time off work [16], lack of transportation [16], language impediments [12, 16, 19], lack of child care [16], no doctor’s recommendation/provider support [15, 17, 18, 20, 21], competing priorities [13, 17], lack of insurance [20, 22], and immigration status [5, 19]. Barriers that prevent women from taking action to screen must be better understood and addressed in order to implement prevention to increase screening, particularly among under-screened women.

Studies on barriers, however, generally focus on women with more regular screening histories and access to healthcare rather than on low-income, under-screened women who are at a notably higher risk of cervical cancer [23]. Data are more limited on racial and ethnic differences in barriers among low-income, under-screened women. Previous studies on barriers to cervical cancer screening have focused on targeting one racial/ethnic group and have not stratified by race and ethnicity [24–26]. It is critical to further understand the barriers that these high-risk women face to inform future screening programs.

Ultimately, increasing screening rates with continuity to follow-up testing and treatment upon indication will decrease the burden of cervical cancer. Intention to screen is a strong indicator of future screening behavior [27, 28]. However, little data have been published on what factors might predict cervical cancer screening intention among low-income under-screened women [11, 29, 30].

To address these data gaps, we examined a large sample of women in North Carolina who are low-income, under- or uninsured, and under-screened according to USPTF guidelines. Our goal is to better understand barriers to screening, stratified by race/ethnicity, among these women at a higher risk of cervical cancer. Predictors of intention to screen among these under-screened women are also explored to inform future screening interventions.

## Methods

Data were obtained from the My Body, My Test Phase 3 (MBMT-3) trial, a two-arm randomized controlled trial (RCT) examining the effect of mailed HPV self-testing on cervical cancer screening completion among under-screened, low-income women [31].

### Participants

Participants were recruited for enrollment between April 2016 and December 2019. Participants were eligible if they were between the ages of 25 and 64, not currently pregnant, had an intact cervix (no history of full hysterectomy), had income ≤250% of the U.S. Federal Poverty Level, were uninsured or enrolled in Medicaid or Medicare, and were living within the catchment area of a trial-associated clinic which included 22 North Carolina counties. In addition, women were eligible only if they self-reported not having a Pap test in 4 years or more, and not having an HPV test in 6 years or more since these women are considered overdue for screening according to USPTF guidelines [3]. Recruitment methods included printed materials (flyers, posters, etc.); online (Facebook, Craigslist) and radio advertisements; referral through the NC United Way 2-1-1 social assistance helpline; and in-person enrollment at community events and trial-associated clinics [31].

### Procedures

Participants completed an eligibility screener in English or Spanish and baseline questionnaire during the MBMT-3 trial. The eligibility screener was conducted by phone to determine trial eligibility, and elicited information on socio-demographic characteristics such as race, ethnicity, education, and health insurance status. We obtained written informed consent. Trial-eligible women received and returned informed consent forms via mail. Participants then completed the baseline questionnaire to collect information on barriers to screening, knowledge, health-seeking behaviors, other socio-demographic characteristics, and attitudes related to cervical cancer and screening. Participants received a phone call providing education on cervical cancer and assistance scheduling an appointment for a free screening at a study-affiliated clinic. Participants received a $25 incentive for completing the baseline survey and $55 for completion of both the follow-up and exit surveys. Approval for this trial was granted by the University of North Carolina Institutional Review Board.

### Measures

Participants were asked several questions about specific barriers to cervical cancer screening. Responses were either based on their level of agreement to each statement using three- or four-point response scales or answers were recorded as “yes,” “no,” “refused,” or “don’t know.” Additionally, perceived barriers to cervical cancer screening were assessed using the following open-ended question: “What are some reasons that you haven’t had a Pap smear recently?” Responses were coded into the following options: “cost,”, “no insurance,” “no time/too busy,” “afraid,” “forgot,” “no doctor” and “don’t know”. The secondary outcome, intention to screen, was assessed through the following question: “How likely are you to get a Pap smear in the next 6 months?" The response scale was “definitely won’t,” “probably won’t,” “probably will,” “definitely will,” and “don’t know.”

The selection of potential predictors was guided by the Health Belief Model [11] and informed by prior literature documenting characteristics associated with barriers and screening behaviors [12, 21]. These variables include demographics (age, marital status, sexual orientation, number of live births), social structure (race/ethnicity, education, employment status, primary language), and resources (income, health insurance status, receipt of social assistance, rurality). Receipt of social assistance refers to any of the following: receipt of food stamps, housing assistance, welfare payments, social security, supplemental security income, and disability payments. Rurality was determined using the 2006 Rural-Urban Commuting Area codes on the basis of participants’ zip codes [32].

Race and ethnicity were ascertained from responses to two survey questions. Responses to “Are you Latina or Hispanic?” were recorded as “yes”, “no”, “refused”, or “don’t know”. Responses to “What is your race or ethnicity?” were recorded in a “mark all that apply” by the interviewer with the following options: “Black or African American”, “White”, “American Indian or Alaska Native”, “Asian”, “Native Hawaiian or Pacific Islander”, “Hispanic/Latina”, “Refused”, “Don’t Know”, including an option to use an open-ended text box to record a race or ethnicity other than the ones listed. We conceptualized race and ethnicity as social constructs that impact individuals’ perceptions, life experiences, and access to and relationship with the healthcare system.

### Data analyses

A total of 759 women were eligible for baseline analyses as described elsewhere [33]. Of these 759 eligible participants, one participant did not self-identify any race or ethnicity, and 48 self-identified as a race or ethnicity other than White, Black, or Hispanic/Latina. Of these 48, 30 self-identified as two or more racial categories, 9 identified as American Indian or Alaskan Native, 6 identified as Asian, and 3 identified as Native Hawaiian or Pacific Islander. In total, 49 participants were excluded from the current analysis due to small sample sizes racial categories. This resulted in a final analytic sample of 710 participants who self-identified as Non-Hispanic White, Non-Hispanic Black or African American, and/or Hispanic or Latina.

Descriptive statistics for demographic and barrier-related questionnaire responses were calculated stratified by race and ethnicity. We calculated chi-square tests to assess differences in sociodemographic characteristics by race (Non-Hispanic White; Non-Hispanic Black or African American; and Hispanic or Latina). Logistic regression was used to assess bivariate associations between race and barriers to cervical cancer screening. Multivariable logistic regression was used to examine the association between demographic factors and barriers with intention to screen within the next six months. Demographic and barrier predictors were considered for inclusion in the final multivariable model based on their conceptual relationship with intention to screen [30, 34] and based on their bivariate association with intention to screen. An alpha level for statistical significance was set at 0.05. Responses to questions about individual barriers to screening were dichotomized from three- or four-point response scales. Variables in the final model were assessed for multicollinearity. All statistical analyses were conducted using SAS software, version 9.4 (Cary, NC) [35].

## Results

### Participants

Participants’ median age was 43 years (White women = 43, Black women = 42, and Hispanic or Latina women = 36) (**Table 1**). Fifty-seven percent of women had attended some college or had a college degree. Most women were uninsured (79%) and had an annual household income under $25,000 (69%). Unemployment was high, reported by over half of White (59%) and Black (56%) women, and 43% of Hispanic or Latina women. Sixty-two percent of White women had an annual household income under $25,000 versus 77% of Black women and 54% of Hispanic or Latina women. Seventy-one percent of women overall intended to receive screening for cervical cancer in the next six months, which was lower in White women (65%) as compared to Black women (76%) and Hispanic or Latina women (72%)(p = 0.007).

**Table 1:** Characteristics of My Body My Test-3 study participants, stratified by race and ethnicity (*n* = 711).

Characteristic	White Non-Hispanic or Latina	Black Non-Hispanic or Latina	Hispanic or Latina
	*n* (%)	*n* (%)	*n* (%)
**Total**	**287**	**356**	**67**
Median age (range)* in years	43 (25–63)	42 (25–64)	36 (25–63)
Education			
High school diploma, GED^‡^, or less	113 (39%)	156 (44%)	36 (54%)
Some college or more	174 (61%)	200 (56%)	31 (46%)
Health insurance*			
Uninsured	236 (82%)	264 (75%)	59 (88%)
Publicly insured^†^	51 (18%)	90 (25%)	8 (12%)
Employment			
Unemployed	167 (59%)	198 (56%)	29 (43%)
Employed, part or full time	114 (41%)	156 (44%)	38 (57%)
Annual household income*			
<$10,000	91 (32%)	135 (38%)	11 (16%)
$10,000 - <$25,000	88 (31%)	141 (40%)	25 (37%)
≥ $25,000	85 (30%)	65 (18%)	25 (37%)
Not reported^§^	23 (8%)	15 (4%)	6 (9%)
Receipt of social assistance* **			
No	162 (57%)	167 (47%)	42 (64%)
Yes	122 (43%)	185 (53%)	24 (36%)
Rurality*^¶^			
Urban	250 (87%)	334 (94%)	61 (91%)
Rural	37 (13%)	22 (6%)	6 (9%)
Intention to screen in the next 6 months*			
Probably will or definitely will	186 (65%)	267 (76%)	48 (72%)
Probably won’t or definitely won’t	99 (35%)	86 (24%)	19 (28%)
Primary language*			
English	280 (99%)	353 (99.7%)	32 (48%)
Not English^||^	2 (1%)	1 (0.3%)	35 (52%)
Marital status*			
Single/never married	111 (39%)	221 (63%)	35 (52%)
Married/living with a partner	65 (23%)	59 (17%)	21 (31%)
Divorced/separated/widowed	106 (38%)	73 (21%)	11 (16%)
At least one visit to healthcare provider in last year			
Yes	195 (68%)	247 (69%)	45 (67%)
No	92 (32%)	109 (31%)	22 (33%)

*Note*. Numbers may sum to less than total sample due to missing or excluded data (Missing Race/Ethnicity n = 47, Age n = 1, Children n = 14, Insurance n = 2, Employment n = 8, Marital Status n = 8, Primary Language n = 7, Intention to Screen n = 5, Social Assistance n = 8, Sex Partners n = 12, Abnormal Pap test n = 87, Rurality n = 3)

* p <0.05.

† Includes Medicaid (n = 142), dual Medicaid/Medicare (n = 12), and Medicare only (n = 5)

‡ General educational development program

§ <250% Federal Poverty Level

^||^ Includes Spanish (n = 35), French (n = 1), Russian (n = 1), and Portuguese (n = 1).

¶Derived from 2006 Rural-Urban Commuting Area (RUCA) codes

**Includes food stamps, housing assistance, welfare payments, social security, Supplemental Security Income, or disability payment

### Reported barriers

**Fig 1:**
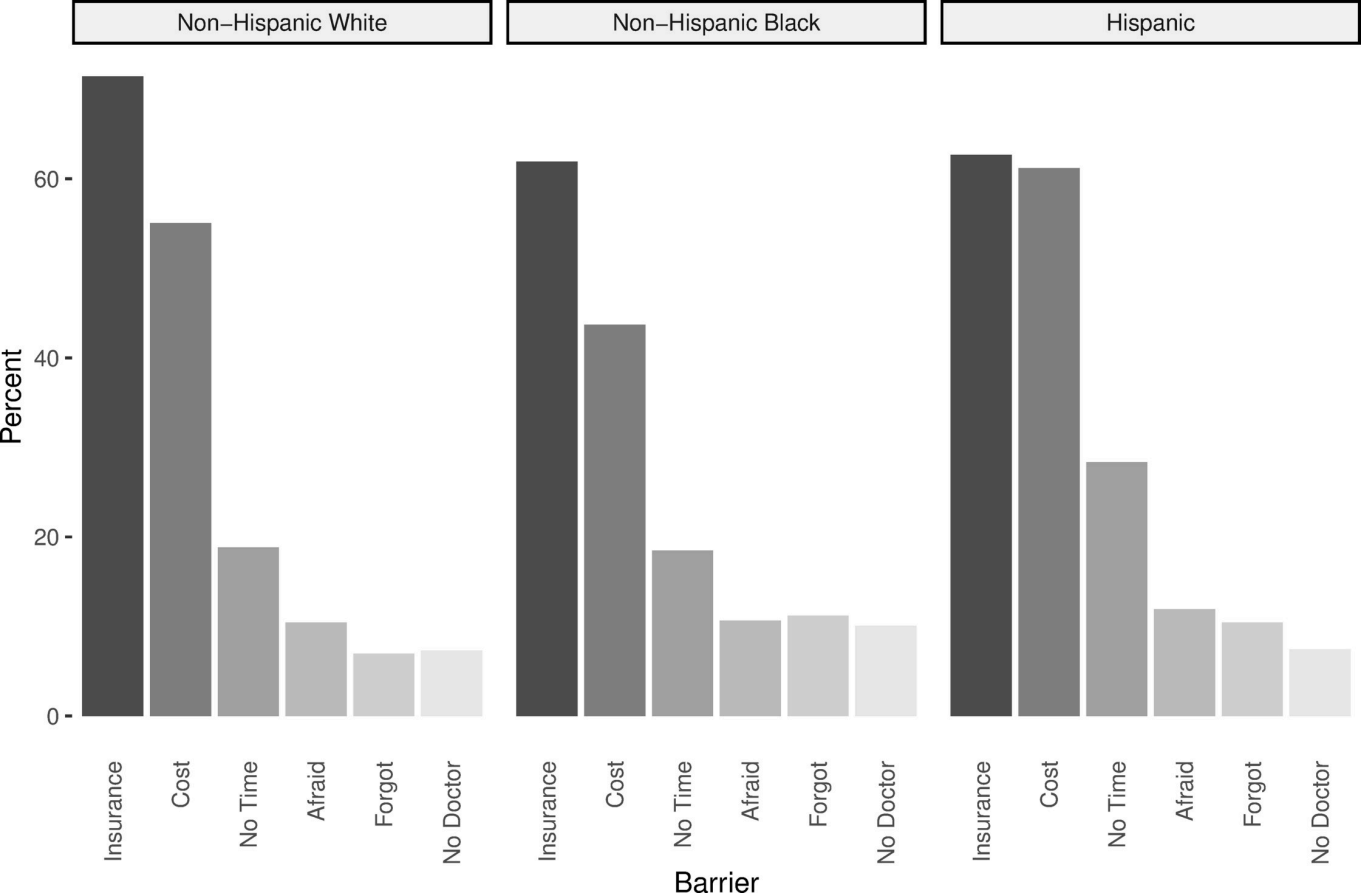
Barriers to cervical cancer screening by race and ethnicity. Responses could be coded as multiple barriers. Other barriers reported less frequently include unsure, don’t need, transportation, didn’t want, missed appointment, not comfortable, not offered, not covered, and spousal opposition.

Reported barriers to screening were similar between racial and ethnic groups (**Fig 1**). The most commonly reported reasons across all three racial and ethnic groups were lack of insurance (White 71%, Black 62%, Hispanic/Latina 63%) and cost (White 55%, Black 44%, Hispanic/Latina 61%). Other commonly reported barriers included having no time (White 19%, Black 19%, Hispanic/Latina 28%), being afraid (White 10%, Black 11%, Hispanic/Latina 12%), forgetting (White 7%, Black 11%, Hispanic/Latina 10%), or not having a doctor (White 7%, Black 10%, Hispanic/Latina 7%).

More than half of all groups surveyed reported five or more barriers to receiving cervical cancer screening: 70% of White women, 63% of Black women and 78% of Hispanic/Latina women (**Table 2**). In terms of observed differences, White women were more likely to report five or more barriers compared to Black women. Hispanic/Latina women were more likely to report having to take time off work to get screened and more likely to have difficulty finding someone to watch their dependents as compared to White women.

**Table 2:** Reported barriers to cervical cancer screening, by race and ethnicity.

	N			*p-value*	*p-value* Hispanic vs Non-Hispanic White
	%			Non-Hispanic Black vs. Non-Hispanic White	
	Non-Hispanic	Non-Hispanic	Hispanic		
	White	Black			
Five or more reported barriers	202/287	224/356	52/67	**0.04**	0.24
	70.4%	62.9%	77.6%		
Doctor has not said to get a Pap smear	209/285	268/351	55/67	0.38	0.14
	73.3%	76.4%	82.1%		
No visit to healthcare provider in last year	86/209	99/268	21/55		
	41.1%	36.9%	38.2%		
At least one visit to healthcare provider in last year	123/209	169/268	34/55		
	58.9%	63.1%	61.8%		
Visit was for preventative care	50/123	98/165	20/34		
	40.6%	59.4%	10.6%		
Does not know about clinics where you can get a free or low-cost Pap smear	189/268	237/331	43/59	0.77	0.72
	70.5%	71.6%	72.9%		
Thinks that it is hard to get cervical cancer screening	162/274	189/347	46/66	0.25	0.12
	59.1%	54.5%	69.7%		
Have to take time off work	90/286	133/351	33/66	0.09	**<0.01**
	31.5%	37.9%	50%		
If so, will lose pay	75/86	115/134	30/33	0.77	0.58
	87.2%	85.8%	90.9%		
Clinics are not open at convenient times	65/254	85/329	17/65	0.95	0.93
	25.6%	25.8%	26.2%		
Hard to find someone to watch dependents	42/180	64/248	20/44	0.56	**<0.01**
	23.3%	25.8%	45.5%		
Too busy to get screened	65/287	73/356	18/67	0.46	0.46
	22.7%	20.2%	26.9%		
Physical or mental health problems preventing access to screening	44/284	48/356	6/67	0.47	0.17
	15.5%	13.5%	9%		
Not comfortable getting screening from a new provider	28/285	26/355	9/67	0.26	0.39
	9.8%	7.3%	13.4%		
Sometimes, always, or often needs help reading instructions, pamphlets, or other written materials from your doctor or pharmacy	8/287	14/356	4/66	0.43	0.20
	2.8%	3.9%	6.1%		

*Note*. Number of reported barriers is the sum of survey question responses indicating a barrier to screening

Another commonly reported barrier across racial and ethnic categories was not being told by a doctor to get a Pap test (White 73%, Black 76%, Hispanic/Latina 82%). Of those who reported this barrier, more than half had at least one visit to a healthcare provider in the last year (White 59%, Black 63%, Hispanic/Latina 62%). Most women did not know about clinics where they could receive a free or low-cost Pap test (White 71%, Black 71%, Hispanic/Latina 73%). Over half of participants also reported that it was hard to get cervical cancer screening (White 59%, Black 55%, Hispanic/Latina 70%).

### Intention to screen

Most surveyed women intended to get a Pap test in the next six months (70.6%). Our multivariable model (**Table 3**) indicated that older women were less likely to intend to screen compared to women ages 25–34 (age 35–49 OR: 0.70 (0.46, 1.08) vs age 25–34; age 50–64 OR: 0.71 (0.44, 1.14) vs age 25–34). Black women were more likely to intend to screen compared to White women (OR: 1.48 (1.02, 2.06)). Reporting that it was not hard to get screening (OR: 1.47 (1.00, 2.15)) and reporting having no time as a barrier to screening (OR: 2.05 (1.23, 3.41)) were associated with higher intention to screen.

**Table 3:** Predictors of intention to screen.

Variable	Intends to screen (%)	Crude OR (95% CI) for intent to screen	Multivariable OR (95% CI)
Age (years)			
25–34	75.6	REF	REF
35–49	68.1	0.69 (0.46, 1.02)	0.70 (0.46, 1.08)
50–64	70.1	0.75 (0.49, 1.16)	0.71 (0.44, 1.14)
Race and Ethnicity			
Non-Hispanic White	65.3	REF	REF
Non-Hispanic Black	75.6	**1.66 (1.18, 2.34)**	**1.48 (1.02, 2.06)**
Hispanic	71.6	1.35 (0.75, 2.41)	1.37 (0.71, 2.68)
Education			
High school diploma or less	75.8	REF	REF
Some college or more	67.5	**0.66 (0.48, 0.93)**	0.71 (0.49, 1.02)
Health Insurance			
Publicly insured	78.2	REF	REF
Uninsured	69.1	**0.62 (0.41, 0.96)**	0.84 (0.52, 1.36)
Doctor said to get a Pap smear			
No	68.8	REF	REF
Yes	77.2	**1.55 (1.04, 2.32)**	1.35 (0.87, 2.11)
Knowledge about free or low-cost clinic			
No	68.1	REF	REF
Yes	78.2	**1.69 (1.14, 2.52)**	1.49 (0.98, 2.26)
Thinks that it is hard to get screening			
Hard	66.8	REF	REF
Not Hard	77.4	**1.70 (1.20, 2.41)**	**1.47 (1.00, 2.15)**
Reported having no time			
No	68.3	REF	REF
Yes	82.5	**2.20 (1.37, 3.54)**	**2.05 (1.23, 3.41)**

Note. Odds ratios are estimating intention vs no intention to screen. Multivariable odds ratios are adjusted by all other variables in the table.

## Discussion

Among this study of 710 under-screened US women, reported barriers to cervical cancer screening showed little variation across race and ethnicity. Having to take time off work and difficulty finding care for dependents were the only barriers which were more common among Hispanic or Latina women than other racial/ethnic groups. Frequently reported barriers were: cost (including lacking knowledge about free or low-cost clinics), not having health insurance, not being told by a doctor to get a cervical cancer screening, and having the perception that getting screened was difficult. Reported intention to get a Pap test was higher among Black women and women who reported not having time to get screening, yet was lower in older women and those who perceived screening to be difficult to get.

Under-screened, low-income women reported many of the same barriers to cervical cancer screening as more frequently screened women. Nearly three-quarters of participants reported a lack of health insurance, a well-documented barrier to screening [24, 25, 29], along with a general lack of knowledge about existing free or low-cost clinics, as barriers. These data suggest that in this population, financial concerns are a major barrier to screening. North Carolina has not expanded Medicaid eligibility, thus many low-income adults remain uninsured [36]. These findings are consistent with many studies that identified cost or lack of insurance as major barriers to cervical cancer screening [14, 15, 22, 37, 38]. Our findings are consistent with results of a previous study using MBMT-3 data, which found that 72% of participants perceived financial barriers to screening and that uninsured women were more likely to perceive screening costs and follow-up treatment costs as barriers [39].

Our study identified that a greater proportion of Hispanic or Latina women reported having to take time off work and difficulty finding care for dependents compared to Black and White women. This is in line with findings from a study of the use of Pap tests among Hispanic and non-Hispanic White women in rural Washington State, US. This study found needing to take time off work was associated with higher screening non-compliance, particularly among highly acculturated Hispanic women [16]. Hispanic or Latina women in our study reported higher rates of employment compared to Black or White women, which could potentially explain these findings.

Not being told to get a Pap test was another commonly reported barrier to screening. This could be an issue of access given that 31% of our participants had not visited a healthcare provider in the past year. Many previous studies have found provider recommendation to be an important facilitator to getting screened [13, 24, 25]. Uninsured women are less likely to receive routine preventive medical care in general [40, 41]. If their only interaction with the healthcare system is when ill or injured, they would be less likely to receive advice or education about cervical cancer screenings.

Our findings highlight the importance of patient outreach and education. Community outreach is necessary to inform uninsured women about the existence of preventative medical screenings and to link them to affordable care. Prior studies have shown that patient navigation can increase screening uptake [42–44]. Helping patients move through the complex healthcare system might make the process less daunting. Programs like the North Carolina Breast and Cervical Cancer Control Program (NC BCCCP) [45] exist to assist this population from screening through diagnosis and follow-up care, but require that women know about the programs and how to access to them.

Despite these reported barriers, most surveyed women reported intention to screen for cervical cancer in the next six months. Older women were less likely to report intention to screen, which is in line with previous studies [29, 30]. Black women were more likely to report intention to screen for cervical cancer compared to White women. Black women and Hispanic/Latina women have higher cervical cancer mortality rates compared to White women [46]. In light of these disparities in cervical cancer incidence and mortality between racial and ethnic groups, it is essential to facilitate turning intention into action.

A major strength of this study is the focus on under-screened and underinsured women. Few studies examine this diverse population even though they are at an especially high risk of cervical cancer [47]. Our large sample of high-risk women allows for an in-depth analysis of barriers to and intent for screening. Among study limitations, participants in the MBMT-3 trial might have been more interested in receiving screening for cervical cancer than other under-screened woman and therefore more likely to respond to study recruitment efforts. As such, participants may have reported different barriers or had greater health seeking behavior than women who were eligible but not interested in participating. As a result, findings from this analysis may not be representative of the general population of under-screened and low-income women. The intention to screen analysis may be subject to social desirability bias if participants reported an intention to screen because they perceived it to be the preferred response. Another limitation of this analysis is the inability to examine reported barriers across broader racial/ethnic categories due to small sample sizes of some racial groups. Previous literature tends to lump these smaller groups into an “Other” category for analyses, which limits the ability to discuss racial/ethnic differences.

Previous studies have shown that intention to screen is positively associated with actual screening uptake, and regular screening leads to better outcomes [2, 27, 28]. Black women generally having worse cervical cancer outcomes compared to White women [46]. However, we found that Black women reported a higher intention to screen and reported fewer barriers. These discrepant findings are likely attributable to inequalities in access to quality care [48–50]. Improved interventions are needed to capitalize on this increased intention to screen among Black women and to assess the most effective facilitators of cervical cancer screenings.

This study identified frequent barriers in this high-risk population regardless of racial and ethnic group. The majority of women in our sample reported cost, lack of insurance, and lack of provider recommendation as barriers. Increasing knowledge of affordable clinics, improving patient education, implementing patient navigation, and expanding access to Medicaid may be effective ways to reduce these barriers and increase screening uptake.
